# Sparing confirmatory testing in primary aldosteronism (SCIPA): a multicenter retrospective diagnostic accuracy study

**DOI:** 10.1186/s12902-024-01638-w

**Published:** 2024-07-08

**Authors:** Albert Macaire C. Ong Lopez, Leo E. Tiu, Diana Collen Dimayuga, Oliver Allan C. Dampil, Erick S. Mendoza, Michael L. Villa, Andrea Marie Macabuag-Oliva

**Affiliations:** 1 Department of Medicine, Section of Endocrinology, Diabetes and Metabolism, St. Luke’s Medical Center- Quezon City, 279 E Rodriguez Sr. Ave, Quezon City, Metro Manila, Philippines; 2 Department of Medicine, Section of Endocrinology, Diabetes and Metabolism, Makati Medical Center, No. 2 Amorsolo Street, Legaspi Village, Makati City, Metro Manila, Philippines; 3 Department of Medicine, Section of Endocrinology, Diabetes and Metabolism, St. Luke’s Medical Center- Global City, Block 16 Lot 7, Crescent District, Rizal Drive corner 32nd Street, Bonifacio Global City, Taguig City, Metro Manila, Philippines

**Keywords:** Primary aldosteronism, Saline infusion test, Plasma aldosterone concentration

## Abstract

**Background:**

The diagnosis of primary aldosteronism (PA) is comprehensive, which includes case-detection testing, case confirmation followed by subtype classification. In certain instances, such as in the setting of spontaneous hypokalemia, suppressed renin activity (PRA) plus plasma aldosterone concentration (PAC) of > 15 ng/dL, one may not proceed with confirmatory tests. However, the quality of evidence behind this approach is very low. This study sought to evaluate the proposed “simplified confirmatory pathway” that can spare confirmatory testing for primary aldosteronism by evaluating the diagnostic performances of the various pre-specified PAC thresholds in combination with findings of suppressed renin and spontaneous hypokalemia.

**Methods:**

This is a multi-center, retrospective diagnostic accuracy cohort-selected cross-sectional study. A total of 133 participants aged 18 years and above underwent saline infusion test between January 2010 to March 2024. The outcome measures comprise of the diagnostic performances of the different index test combinations (baseline PAC, baseline PRA and presence of spontaneous hypokalemia): sensitivity, specificity, negative predictive value, positive predictive value, positive likelihood ratio, negative likelihood ratio, and diagnostic accuracy. Data analysis was performed using SPSS 29.0.1.0 & MedCalc 20.218.

**Results:**

Of the 133 patients who underwent saline infusion test, 88 (66.17%) were diagnosed with PA. A PAC of > 25 ng/dL plus PRA < 1.0 ng/dL/hr with spontaneous hypokalemia showed the highest specificity at 100% (95% CI 90.51%, 100.00%) and positive predictive value at 100% (85.18 – 100.00%). The minimum acceptable combination criteria were determined to be a PAC of > 20 ng/dL plus PRA < 0.6 ng/dL/hr, and presence of spontaneous hypokalemia. It has high specificity (94.59%; 95% CI 81.81%, 99.34%), positive predictive value (93.55%, 95% CI 78.49%, 98.29%), and moderate positive likelihood ratio (LR+) (6.39, 95% CI 1.61, 25.38)

**Conclusion:**

A hypertensive patient with spontaneous hypokalemia and screening findings of PAC > 20 ng/dL and suppressed PRA of < 0.6 ng/ml/hr, may be classified as “overt primary aldosteronism confirmed” and may not need to proceed with dynamic confirmatory testing.

**Protocol registration number:**

SRCTN34186253

## Introduction

Primary aldosteronism is one of the most common causes of secondary hypertension. It is often unrecognized, especially if mild in severity. In a systematic review totaling 42,510 patients, prevalence estimates ranged from 3.2% to 12.7% in primary care and from 1% to 29.8% in referral centers [1]. The prevalence was identified to be higher in patients with new onset diabetes mellitus. In a study performed in an outpatient department which included 256 individuals with hypertension and diabetes mellitus, 49 (19%) were diagnosed with primary aldosteronism [2].

The biochemical profile of primary aldosteronism typically shows an elevated plasma aldosterone, suppressed renin, and occasionally low potassium levels. Based on a retrospective study of a French hypertensive population which included 173 patients, baseline plasma aldosterone concentration was 3-fold higher among those with aldosterone producing adenoma and idiopathic aldosteronism than in those with essential hypertension (*p* <0.0001) [3]. Ninety-one percent of patients with aldosterone producing adenoma had baseline plasma aldosterone concentration above 550 pmol/l (>19.83 ng/dL) as compared to only 14% in subjects with essential hypertension [3]. Direct renin concentrations were likewise lower in patients with aldosterone producing adenoma and idiopathic aldosteronism compared to patients with essential hypertension (*p*< 0.001 and *p* < 0.02, respectively) [3]. Moreover, 82.9 % of patients with aldosterone producing adenoma are hypokalemic versus only 20.2% for patients with essential hypertension [3].

Correctly diagnosing primary aldosteronism entails an initial case-detection testing which involves obtaining a morning blood sample of plasma aldosterone concentration, plasma renin activity or plasma renin concentration in a seated position. After case detection, according to the Endocrine Society Guidelines 2016, patients who had a positive aldosterone renin ratio (ARR) result should undergo one or more confirmatory tests to confirm or exclude primary aldosteronism [4]. These confirmatory tests include the saline infusion test, captopril challenge test, oral sodium loading test and the fludrocortisone suppression test. Despite its many benefits, confirmatory testing may at times be costly, time-consuming, and inconvenient.

There are however some exceptions to the requirement for confirmatory testing. According to the Endocrine Society Guidelines 2016, in the setting of very low plasma renin levels, a plasma aldosterone concentration (PAC) of >20 ng/dL with spontaneous hypokalemia, one does not need to proceed with further confirmatory testing [4]. Moreover, in an updated approach to the diagnosis of primary aldosteronism, a suppressed renin activity (PRA) of at least less than 1.0 ng/mL/h (ideally plasma renin activity < 0.6 ng/mL/h or plasma renin concentration < 5 mU/L), plasma aldosterone concentration greater than 15 ng/dL and a high pretest probability consisting of resistant hypertension and/or hypokalemia, an “overtly positive screen” is detected and no further dynamic testing is required [5]. However, these recommendations have very low-quality evidence. The suitable setting for sparing confirmatory tests is still under debate and requires more investigation.

This study sought to evaluate the proposed “simplified confirmatory pathway”, which will exclude the need for dynamic confirmatory testing for primary aldosteronism and analyze the findings in line with the recent Endocrine Society clinical practice guideline criteria and recommendations. Other specific objectives include: a) to describe the sociodemographic, clinical and laboratory characteristics of patients who underwent the saline infusion test, b) to elucidate the subtype classification proportion of patients with positive saline infusion test with unilateral aldosteronoma, bilateral adrenal hyperplasia, or adrenocortical carcinoma on adrenal CT and/or surgical pathology, and c) to determine the diagnostic performances of various combination criteria including the baseline plasma aldosterone concentration (PAC), plasma renin activity (PRA) and presence of spontaneous hypokalemia.

## Methodology

This study was conducted in accordance with the 2015 STARD statement (Standards for Reporting of Diagnostic Accuracy Studies) [6].

### Study design and population

This study was conducted as a retrospective diagnostic accuracy cohort-selected cross-sectional study [7] at multiple outpatient referral centers namely – St. Luke’s Medical Center-Quezon City, St. Luke’s Medical Center-Bonifacio Global City, and Makati Medical Center.

Included in this study were all patients 18 years old and above who underwent saline suppression testing between January 2010 to March 2024 whose formal conduct of this test was only proceeded based on the following primary aldosteronism positive screening criteria: an aldosterone-renin ratio (ARR) of ≥ 20 or a suppressed plasma renin activity (PRA) of less than 1.0 ng/ml/hr with or without plasma aldosterone concentration (PAC) levels of > 5 ng/dL.

Individuals who underwent screening for primary aldosteronism (baseline PAC and PRA) were cases of uncontrolled blood pressure of > 140/90 mmHg despite on 3 anti-hypertensive regimens; or controlled blood pressure of < 140/90 on at least 4 anti-hypertensive drugs; presence of spontaneous hypokalemia defined as serum potassium < 3.5 mEq/L without drug interference; hypertensive patients with first degree relative of PA; and functional work-up for adrenal incidentaloma.

Conversely, those who were unable to complete the saline suppression test or did not comply with the saline infusion protocol (pre-saline potassium levels must be at least 3.5 mEq/L; and no intake of mineralocorticoid receptor antagonists for past 30 days) were excluded. If the patient is already receiving potassium supplementation and the potassium levels are adequately replete (≥3.5 mEq/L), they remain included in the saline infusion testing.

The main source of lists for subject screening and selection were obtained from the logbook and other electronic registries from the respective diabetes, thyroid, and endocrine centers.

### Data collection

Relevant clinical and laboratory data were retrieved from eligible patients who underwent saline infusion testing via the electronic medical records. Sociodemographic and clinical characteristics such as the presence of hypertension, diabetes mellitus and eGFR stage were reviewed. Pertinent laboratory blood exams comprised of the baseline plasma renin activity, plasma aldosterone concentration, and serum potassium level, which were used initially in the screening process for primary aldosteronism prior to confirmatory testing if needed depending on the screening results. Likewise, abdominal CT-scan findings and other surgical and/or histopathology data were acquired. Both pre-infusion and post-infusion plasma aldosterone concentration data were also collected as mandatory criteria to determine the saline infusion test results.

### Test methods

The index test mainly investigated in this study was the combination of baseline plasma aldosterone concentration (PAC) at several pre-specified cutoffs points (> 10, >15, >20, & >25 ng/dL respectively), with suppressed baseline plasma renin activity (PRA) (at least less than 1.0 ng/mL/hr and less than 0.6 ng/ml/hr) and presence of spontaneous hypokalemia (defined as serum potassium < 3.5 mmol/L without drug interference such as diuretic use) for which the initial variable groupings were then reclassified into dichotomous categories (yes or no) depending on the stated cutoff or threshold.

For the reference standard, the saline infusion test (SIT) was used for confirming the presence or absence of primary aldosteronism. Prior to performing the said test, patients were required not to have any intake of potassium-sparing diuretic or any diuretic for at least 4 weeks. Potassium levels should also be within normal limits. Confirmatory testing was conducted in accordance with a pre-specified institution-based protocol. Patients remained in a recumbent position for at least 1 hour prior and during the infusion of 0.9% sodium chloride at a rate of 500 ml per hour over 4 hours for a total of 2 liters. Blood samples for plasma aldosterone concentration and serum potassium were drawn at time zero and after 4 hours. This saline infusion test protocol was similar across the involved hospital institutions in this study. Post-saline infusion, none of the tested individuals developed any complications or adverse effects.

A positive test result for primary aldosteronism was initially defined as post-infusion plasma aldosterone levels of > 10 ng/dL (280 nmol/L); whereas those with post-infusion plasma aldosterone levels of <5 ng/dL (140 pmol/L) are unlikely to have a diagnosis of primary aldosteronism. For indeterminate values between 5 and 10 ng/dL, a threshold of 6.8 ng/dL (190 pmol/L) was used [4] in reclassifying a positive test for primary aldosteronism. The Endocrine Society Guidelines 2016 states that a cutoff of 6.8 ng/dL offers the “best trade-off between sensitivity and specificity” [4]. Thus, based on this recommendation, a final cutoff of post-infused plasma aldosterone levels ≥ 6.8 ng/dL was categorized as confirmed positive.

Since this was a retrospective study, the data assessor had access to all patient information, baseline medical tests, and the saline infusion test results. Nevertheless, the final classification of a positive (disease confirmed) or negative (disease excluded) primary aldosteronism as based on the reference standard was not guided or influenced by the index test results or the baseline values. The interpretation of the results was followed strictly and objectively depending on the pre- and post-saline infusion plasma aldosterone concentration values as detailed above.

### Outcome measures

Measures of diagnostic accuracy which include the sensitivity, specificity, positive predictive value, negative predictive value, positive likelihood ratio, negative likelihood ratio and the diagnostic accuracy of the different combination set of criteria were evaluated to determine its discriminative and predictive potential.

### Data analysis

Descriptive statistics were used to summarize the general and clinical characteristics of the participants. Frequencies and percentages were presented for categorical nominal/ordinal data, while the mean and standard deviation were used for normally distributed continuous interval/ratio variables, and median and interquartile range for non-normally distributed continuous interval/ratio variables.

Differences between two groups (PA vs non-PA) were assessed by χ^2^ or Fisher’s exact test for dichotomous categorical variable, Mann-Whitney U test for non-normally distributed numerical variables, and Student’s t test for normally distributed data, as appropriate.

The diagnostic accuracy measures were reported with their 95% confidence intervals to assess the diagnostic performances of the different combinations of plasma aldosterone concentration (PAC), plasma renin activity (PRA) and serum potassium (K+) in detecting primary aldosteronism. Null hypothesis was rejected at 0.05 α-level of significance. IBM SPSS version 29.0.1.0 (SPSS Inc., Chicago, IL, USA) and MedCalc 20.218 (MedCalc Software, Ostend, Belgium) were used for statistical analysis.

### Handling of missing data

Missing data were neither replaced nor imputed. A total of 3 patients had missing serum potassium, 8 had missing baseline PRA, and one had missing baseline PAC data. Thus, such patients were excluded during the main analysis stage.

### Sample size estimation

The sample size calculation for sensitivity and specificity of a single diagnostic test with a binary outcome according to Buderer et. al. was performed [8]. A total sample size of 133 participants was achieved at a confidence level of 95%, precision of 0.109, with an estimated crude prevalence rate of 26% as based on the diagnostic accuracy study by Song et al. [9], and assuming 100% specificity and 12% sensitivity as referenced from the validation cohort saline infusion test dataset of Wang et. al [10].

### Assay methods and reference ranges

For the baseline PAC and PRA screening laboratories, not all blood extractions were performed at the centers included in this study as some initial results were brought in already by the patients at the specialized clinics. However, in majority of screening cases, and in all patients who underwent saline suppression testing at the specialized centers, plasma aldosterone levels (ng/dL) were measured by radioimmunoassay (Aldosterone RIA kit, IM1664, Beckman Coulter Inc; Brea, Ca, USA in St. Luke’s Medical Center; Aldosterone RIA CT, R-CW-100; DiaSource; Louvain-la-Neuve, Belgium in Makati Medical Center). The limit of detection for the former assay was 2.58 ng/dL, while the analytical sensitivity of the latter assay was 0.14 ng/dL. The conventional reference ranges on supine position were 6.41-29.98 ng/dL for the Aldosterone RIA Beckman Kit and 1-16 ng/dL for the Aldosterone RIA CT DiaSource Kit. For the upright position, the ranges were 7.25-36.17 ng/dL and 3.5-30 ng/dL, respectively. Also, the plasma renin activity was measured by radioimmunoassay (Angiotensin I RIA kit, IM 3518, Beckman Coulter Inc; Brea, Ca, USA for both institutions) with an analytical sensitivity of 0.07 ng/ml and a functional sensitivity of 0.20 ng/ml. The expected reference values were 0.30 – 1.90 ng/ml/hr for early morning, supine position, and 0.48 – 4.88 ng/ml/hr for upright position.

## Results

### Flow of participants

**Fig. 1:**
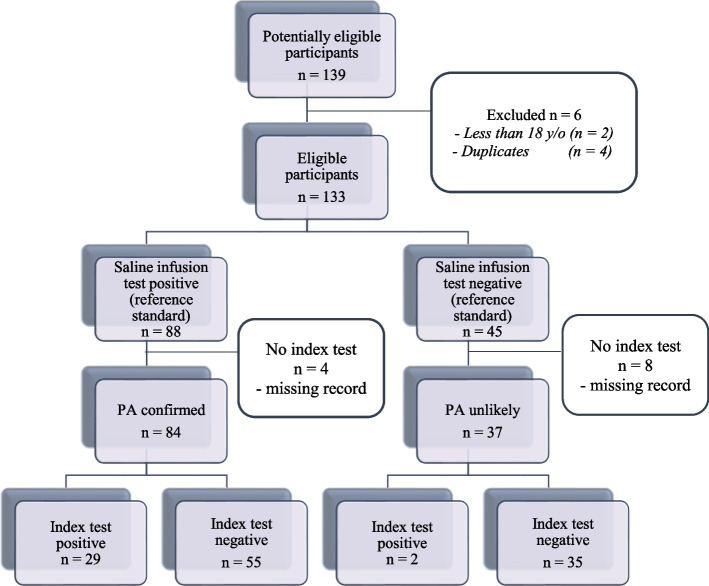
Flow of participants**.** Legend: PA – primary aldosteronism, PAC – plasma aldosterone concentration, PRA – plasma renin activity, Index test criteria – PAC > 20 ng/dL plus PRA < 0.6 ng/ml/hr with spontaneous hypokalemia

A total of 133 eligible patients who had saline infusion testing between January 2010 to March 2024 were included in this study. Figure 1 shows the flow of participants throughout the study.

### Clinical characteristics of the subjects

Table 1 presents the clinical characteristics of patients with primary aldosteronism (PA) and without primary aldosteronism (non-PA). Among the 133 included patients who had confirmatory saline infusion test (SIT), 88 (66.17%) were diagnosed with PA and 45 (33.83%) did not have primary aldosteronism.

**Table 1:** Clinical characteristics between primary aldosteronism (PA) and non-PA patients (*n*=133)

	**All** **(*n*=133)**	**PA** **(*n*=88)**	**Non-PA** **(*n*=45)**	***p*-value**
	**Mean ± SD; Median (IQR); Frequency (%)**			
Age**,** years	49.83±11.26	48.94±12.18	51.56±9.08	0.166*
Sex at birth				0.635**
Male	45 (33.8)	31 (35.2)	14 (31.1)	
Female	88 (66.2)	57 (64.8)	31 (68.9)	
Hypertension (*n*=123)	113 (91.9)	79 (94.0)	34 (87.2)	.286†
Diabetes mellitus (*n*=120)	37 (30.8)	27 (33.3)	10 (25.6)	.393**
eGFR ml/min/1.73 m^2^ (based from 2021 CKD-EPI) (*n*=111)				.625**
≥ 90	64 (57.7)	40 (54.1)	24 (64.9)	
60-89	32 (28.8)	23 (31.1)	9 (24.3)	
45-59	13 (11.7)	10 (13.5)	3 (8.1)	
30-44	2 (1.8)	1 (1.4)	1 (2.7)	
Spontaneous hypokalemia (*n*=129)	79 (61.2)	57 (65.5)	22 (52.4)	.151**
Baseline plasma aldosterone concentration (PAC), ng/dL	18.43 (11.74-29.40)	20.47 (13.85-33.78)	13.25 (9.49-23.21)	0.002‡
Baseline plasma renin activity (PRA), ng/ml/h	0.28 (0.15-0.52)	0.26 (0.16-0.51)	0.31 (0.15-0.56)	0.452‡
Baseline Aldosterone-renin ratio (ARR), ng/dL/ng/ml/h	61.95 (31.74-142.61)	80.62 (38.72-177.27)	39.80 (29.86-84.42)	0.010‡

Statistical tests used

^*^Independent t-test

^**^Pearson Chi-Square test

^†^Fisher’s Exact test

^‡^Mann-Whitney U test

The mean age of the participants was 49.83±11.26 years, and 88 patients (66.2%) were female. In terms of co-morbidities, 123 participants (91.9%) were hypertensive, while 37 of them (30.8%) were diagnosed with diabetes mellitus. Most of these patients (86.5%) have eGFR of > 60 ml/min/1.73 m^2^ .

Between patients with PA and without PA, there was insufficient evidence to detect any statistically significant differences in age, sex distribution, presence of hypertension, diabetes mellitus, or eGFR stage between the two groups (*p*>0.05). The prevalence of spontaneous hypokalemia was higher in patients with PA (65.5%) compared patients without PA (52.4%), but the difference was not statistically significant (*p*=0.186).

Noteworthy was a statistically significant difference in the baseline plasma aldosterone concentration between the two groups, with higher levels observed in patients with PA (median 20.47 ng/dL, IQR: 13.85 – 33.78) compared to patients without PA (median 13.25 ng/dL, IQR: 9.49 – 23.21, *p* = 0.002). In contrast, there was no statistically significant difference detected in the baseline plasma renin activity or aldosterone-renin ratio between the two groups (*p*>0.05).

Table 2 presents the CT-scan findings and histopathology in patients who had a positive saline infusion test (SIT). Among the 88 patients, 72 (82.8%) had abdominal CT scan results, and among them, 5 (6.94%) had bilateral nodules, 52 (72.22%) had unilateral nodules, and 15 (20.83%) had no discrete nodules seen. None of the patients underwent adrenal vein sampling for laterality.

**Table 2:** CT-scan findings and histopathology in patients with positive saline infusion test (*n*=88)

	**Frequency (%)**
Abdominal CT done	72 (82.8)
Laterality	
Bilateral nodule	5 (6.94)
Unilateral nodule	52 (72.22)
No discrete nodule seen	15 (20.83)
Surgery done	20 (22.7)
Histopathology	
Adrenocortical adenoma	14 (73.68)
Adrenocortical carcinoma	1 (5.26)
Adrenocortical neoplasm^a^	2 (10.53)
Adrenocortical hyperplasia Endothelial cyst	1 (5.26) 1 (5.26)

^a^Indeterminate findings (adenoma vs carcinoma)

A unilateral adrenalectomy was performed in 20 patients (22.7%). Of these, histopathological examination revealed that 14 patients (73.68%) had adrenocortical adenoma, one (5.26%) had adrenocortical carcinoma, one (5.26%) had adrenocortical hyperplasia, 2 (10.53%) had adrenocortical neoplasm, and one patient (5.26%) had an endothelial cyst.

### Estimates of diagnostic accuracy and precision

The results (Table 3) showed that combinations A4 (PAC > 25 ng/dL + PRA < 1.0 ng/dL/hr + spontaneous hypokalemia) and A8 (PAC > 25 ng/dL + PRA < 0.6 ng/dL/hr + spontaneous hypokalemia) both showed the highest specificity of 100% (95% CI 90.51%, 100.00%) and positive predictive value (PPV) of 100% (95% CI 85.18%, 100.00%). Also, combinations A2 (PAC > 15 ng/dL + PRA < 1 ng/dL/hr + spontaneous hypokalemia) and A6 (PAC > 15 ng/dL + PRA < 0.6 ng/dL/hr + spontaneous hypokalemia) demonstrated the highest diagnostic accuracy of 56.20% (95% CI 46.89, 65.20%).

**Table 3:** Diagnostic performance of laboratory findings for primary aldosteronism

	**Sensitivity, %**	**Specificity, %**	**PPV, %**	**NPV, %**	**LR+**	**LR-**	**Accuracy, %**
	**Estimate (95% CI)**						
**3 combinations**							
A1: PAC > 10 ng/dL + PRA < 1.0 ng/dL/hr + spont. hypoK	48.81% (37.74 – 59.96%)	70.27% (53.02 – 84.13%)	78.85% (68.44 – 86.50%)	37.68% (31.02 – 44.84%)	1.64 (0.96 - 2.82)	0.73 (0.54 – 0.98)	55.37% (46.06 - 64.41%)
A2: PAC > 15 ng/dL + PRA < 1.0 ng/dL/hr + spont. hypoK	44.05% (33.22 - 55.30%)	83.78% (67.99 – 93.81%)	86.05% (74.04 – 93.02%)	39.74% (34.23 – 45.53%)	2.72 (1.26 – 5.87)	0.67 (0.53 - 0.85)	56.20% (46.89 -65.20%)
A3: PAC > 20 ng/dL + PRA < 1.0 ng/dL/hr + spont. hypoK	34.52% (24.48 – 45.69%)	89.19% (74.58 - 96.97%)	87.88% (73.30 – 95.04%)	37.50% (33.13 – 42.09%)	3.19 (1.21 -8.43)	0.73 (0.61 - 0.89)	51.24% (41.99 - 60.43%)
A4: PAC > 25 ng/dL + PRA < 1.0 ng/dL/hr + spont. hypoK	27.38% (18.21 - 38.20%)	100.00% (90.51 - 100.00%)	100.00% (85.18 – 100.00%)	37.76% (34.72 – 40.89%)	-	0.73 (0.64 - 0.83)	49.59% (40.37 – 58.82%)
A5: PAC > 10 ng/dL + PRA < 0.6 ng/dL/hr + spont. hypoK	46.43% (35.47 – 57.65%)	72.97% (55.88 - 86.21%)	79.59% (68.65 – 87.41%)	37.50% (31.21 – 44.24%)	1.72 (0.96 – 3.06)	0.73 (0.56 – 0.97)	54.55% (45.24 – 63.62%)
A6: PAC > 15 ng/dL + PRA < 0.6 ng/dL/hr + spont. hypoK	41.67% (31.00 – 52.94%)	89.19% (74.58 – 96.97%)	89.74% (77.02 -95.81%)	40.24% (35.25 -45.45%)	3.85 (1.48 – 10.06)	0.65 (0.53 - 0.81)	56.20% (46.89 – 65.20%)
A7: PAC > 20 ng/dL + PRA < 0.6 ng/dL/hr + spont. hypoK	34.52% (24.48 – 45.69%)	94.59% (81.81 - 99.34%)	93.55% (78.49 – 98.29%)	38.89% (34.86 – 43.08%)	6.39 (1.61 – 25.38)	0.69 (0.58 - 0.82)	52.89% (43.61 - 62.03%)
A8: PAC > 25 ng/dL + PRA < 0.6 ng/dL/hr + spont. hypoK	27.38% (18.21 - 38.20%)	100.00% (90.51 - 100.00%)	100.00% (85.18 – 100.00%)	37.76% (34.72 – 40.89%)	-	0.73 (0.64 - 0.83)	49.59% (40.37 – 58.82%)
**2 Combinations**							
B1: PAC > 10 ng/dL + PRA < 1.0 ng/dL/hr	79.76% (69.59 - 87.75%)	35.90% (21.20 – 52.82%)	72.83% (67.42 – 77.63%)	45.16% (31.20 – 59.93%)	1.24 (0.96 - 1.61)	0.56 (0.31 - 1.02)	65.85% (56.76 – 74.16%)
B2: PAC > 15 ng/dL + PRA < 1.0 ng/dL/hr	66.67% (55.54 – 76.58%)	61.54% (44.62 – 76.64%)	78.87% (70.94 – 85.10%)	46.15% (36.69 – 55.90%)	1.73 (1.13 - 2.65)	0.54 (0.37 - 0.80)	65.04% (55.92 -73.42%)
B3: PAC > 20 ng/dL + PRA < 1.0 ng/dL/hr	48.81% (37.74 – 59.96%)	79.49% (63.54 – 90.70%)	83.67% (72.68 – 90.80%)	41.89% (35.66 – 48.39%)	2.38 (1.24 - 4.58)	0.64 (0.50 - 0.84)	58.54% (49.31 - 67.35%)
B4: PAC > 25 ng/dL + PRA < 1.0 ng/dL/hr	41.67% (31.00 – 52.94%)	89.74% (75.78 – 97.13%)	89.74% (76.97 – 95.82%)	41.67% (36.68 – 46.83%)	4.06 (1.55 – 10.63)	0.65 (0.53 - 0.80)	56.91% (47.68 - 65.80%)
B5: PAC > 10 ng/dL + PRA < 0.6 ng/dL/hr	67.86% (56.78 – 77.64%)	43.59% (27.81 – 60.38%)	72.15% (65.46 – 77.98%)	38.64% (28.17 – 50.27%)	1.20 (0.88 - 1.64)	0.74 (0.46 - 1.18)	60.16% (50.95- 68.88%)
B6: PAC > 15 ng/dL + PRA < 0.6 ng/dL/hr	54.76% (43.52 – 65.66%)	69.23% (52.43 – 82.98%)	79.31% (69.73 – 86.45%)	41.54% (34.15 – 49.33%)	1.78 (1.07 - 2.96)	0.65 (0.48 – 0.90)	59.35% (50.12 – 68.11%)
B7: PAC > 20 ng/dL + PRA < 0.6 ng/dL/hr	41.67% (31.00 – 52.94%)	84.62% (69.47 – 94.14%)	85.37% (72.82 – 92.70%)	40.24% (34.97 – 45.75%)	2.71 (1.24 - 5.90)	0.69 (0.55 - 0.86)	55.28% (46.06 - 64.25%)
B8: PAC > 25 ng/dL + PRA < 0.6 ng/dL/hr	32.14% (22.36 – 43.22%)	97.44% (86.52 - 99.94%)	96.43% (79.19 - 99.48%)	40.00% (36.33 – 43.79%)	12.54 (1.77 – 88.95)	0.70 (0.60 - 0.81)	52.85% (43.64 - 61.91%)

Legend: *PAC* plasma aldosterone concentration, *PRA* plasma renin activity, Spont. hypoK – spontaneous hypokalemia, *PPV* positive predictive value, *NPV* negative predictive value, *LR+* – positive likelihood ratio, *LR* negative likelihood ratio

Amongst the overall diagnostic performance estimates, the most balanced and satisfactory criteria to spare confirmatory testing for primary aldosteronism appears to be A7, which consists of PAC > 20 ng/dL plus PRA < 0.6 ng/dL/hr, and presence of spontaneous hypokalemia. The following combination demonstrates the following results: sensitivity: 34.52% (95% CI 24.48% , 45.69%), specificity: 94.59% (95% CI 81.81%, 99.34%), positive predictive value (PPV): 93.55% (95% CI 78.49%, 98.29%), negative predictive value (NPV): 38.89% (95% CI 34.86%, 43.08%), positive likelihood ratio (LR+): 6.39 (95% CI 1.61, 25.38), negative likelihood ratio (LR-): 0.69 (95% CI 0.58, 0.82), and diagnostic accuracy: 52.89% (95% CI 43.61%, 62.03%). It has high specificity and positive predictive value, with a modest positive likelihood ratio. This indicates that screening positive for this combination criteria is reasonably reliable for confirming primary aldosteronism.

## Discussion

The saline infusion test is one of the confirmatory tests commonly used in our setting because it is the most accessible among all the confirmatory tests; however, it may still be cumbersome, time-consuming, and relatively expensive especially in developing countries. Thus, in patients with a profile of resistant hypertension and spontaneous hypokalemia, no further dynamic testing is needed if the screening showed a plasma aldosterone concentration greater than 15 ng/dL and an ideal suppressed renin activity of less than 0.6 ng/ml/hr as based on the 2020 Endocrine Society Review [5]. Nevertheless, the PAC cutoff in the 2016 Endocrine Society clinical practice guideline is slightly higher at 20 ng/dL [4].

The key finding in this study showed that a plasma aldosterone concentration of > 20 ng/dL, plasma renin activity of < 0.6 ng/ml/hr and presence of spontaneous hypokalemia had an overall satisfactory diagnostic performance as compared with other combination parameters. It has relatively high specificity and positive predictive value with modest positive likelihood ratio. This analysis demonstrated that our findings are consistent with the 2016 Endocrine Society recommended approach to primary aldosteronism which specify that patients with plasma renin activity below detection levels combined with an aldosterone concentration > 20 ng/dl and spontaneous hypokalemia can bypass confirmatory testing.

Different cut-off points for plasma aldosterone concentration were suggested by various studies. In a retrospective cross-sectional study from a single referral center in Japan, 327 patients with abnormal ARR who underwent confirmatory captopril challenge test were recruited. The authors demonstrated that 100% (26 patients) of those who had PAC of 20 – 30 ng/dL with spontaneous hypokalemia can be classified as positive for primary aldosteronism [11]. Similarly, a large Chinese cohort study of 784 hypertensive patients in which a PAC of >20ng/dl, PRC<2.5 μIU/ml and spontaneous hypokalemia offered the optimal trade-off between improved sensitivity 36% (32-0.40) and high specificity 100% (97-100) to spare confirmatory testing [10]. Furthermore, in another large multi-institutional, retrospective, cohort study conducted in Japan, a total of 2,256 patients (using captopril challenge test), and/or 1,184 patients (using saline infusion test) were studied. The authors concluded that confirmatory testing could be omitted in patients with baseline PAC ≥ 30.85 ng/dl in the presence of baseline PRA ≤ 0.6 ng/ml/hr with 100% specificity [12]. The variance in PAC cut-offs observed in the previous reports can be related to methodological heterogeneity such as a) cohort size difference; b) the use of fludrocortisone suppression test or saline infusion test [10], captopril challenge test only [11]; saline infusion test and/or captopril challenge test [12] as confirmatory testing; and c) variability in the assay methods and its performance (radioimmunoassay or chemiluminescent enzyme immunoassay). None of these previous studies have the same kit used in our study thus the reference ranges of the PAC and PRA may have slight differences.

In the clinical setting, sparing confirmatory testing for primary aldosteronism using the recommended Endocrine Society criteria can reduce unnecessary cost and is especially useful for places with limited resources. Nevertheless, this criterion also has its shortcomings. While it can reliably confirm the subject as truly having the disease when the result is positive, a negative test result will not fully rule out the condition (Table 4). Therefore, if the criterion is not satisfied, one must still proceed with any of the confirmatory tests available.

**Table 4:** Sparing confirmatory testing rate

**3 combinations**	**Sparing confirmatory testing rate**
A1: PAC > 10 ng/dL + PRA < 1.0 ng/dL/hr + spont. hypoK	48.8% (41/84)
A2: PAC > 15 ng/dL + PRA < 1.0 ng/dL/hr + spont. hypoK	44.0% (37/84)
A3: PAC > 20 ng/dL + PRA < 1.0 ng/dL/hr + spont. hypoK	34.5% (29/84)
A4: PAC > 25 ng/dL + PRA < 1.0 ng/dL/hr + spont. hypoK	27.4% (23/84)
A5: PAC > 10 ng/dL + PRA < 0.6 ng/dL/hr + spont. hypoK	46.4% (39/84)
A6: PAC > 15 ng/dL + PRA < 0.6 ng/dL/hr + spont. hypoK	41.7% (35/84)
A7: PAC > 20 ng/dL + PRA < 0.6 ng/dL/hr + spont. hypoK	34.5% (29/84)
A8: PAC > 25 ng/dL + PRA < 0.6 ng/dL/hr + spont. hypoK	27.4% (23/84)
**2 combinations**	
B1: PAC > 10 ng/dL + PRA < 1.0 ng/dL/hr	79.8% (67/84)
B2: PAC > 15 ng/dL + PRA < 1.0 ng/dL/hr	66.7% (56/84)
B3: PAC > 20 ng/dL + PRA < 1.0 ng/dL/hr	48.8% (41/84)
B4: PAC > 25 ng/dL + PRA < 1.0 ng/dL/hr	41.7% (35/84)
B5: PAC > 10 ng/dL + PRA < 0.6 ng/dL/hr	67.9% (57/84)
B6: PAC > 15 ng/dL + PRA < 0.6 ng/dL/hr	54.8% (46/84)
B7: PAC > 20 ng/dL + PRA < 0.6 ng/dL/hr	41.7% (35/84)
B8: PAC > 25 ng/dL + PRA < 0.6 ng/dL/hr	32.1% (27/84)

Legend: *PAC* plasma aldosterone concentration, *PRA* plasma renin activity, Spont. hypoK – spontaneous hypokalemia

^*^The sparing confirmatory testing rate was defined as the number of patients who can skip the confirmatory test for PA based on each criterion divided by the number of patients who were diagnosed as PA based on SIT confirmatory test.

The main strength of this study is that it was able to validate the recommendations of the guideline criteria and confirmed the suggested cutoff value. In addition, this was conducted in the setting of a developing country with involvement of multiple hospital centers hence reinforcing its clinical and practical applicability.

Our study has certain limitations. Firstly, given its retrospective cross-sectional study design, not all eligible patients may have been identified, leading to missing data points which might have affected the quality of the data [6]. Secondly, the saline infusion test is only available in a few specialized centers in the country; consequently, is rarely done. Thus, despite exhausting all the available databases, the study included relatively fewer participants which may lead to widened confidence interval with imprecise estimates and thus affect generalizability of results. Additionally, the global diagnostic accuracy measure in the test results was relatively low (<60%), which is rather expected given the higher disease prevalence (68%) in this study. Those who are sent for saline infusion testing typically are suspected of having the condition; hence an unintended consequence of selection bias may be introduced. Intra-variability in the sensitivity, accuracy and precision of the assay methods was also encountered as different assay kits were used by different hospital institutions which may affect external validity.

Finally, the assessors of the reference standard have had access to the index test results which may introduce bias that may distort measures of test accuracy especially in situations entailing subjective interpretation [6]. Nonetheless, the definitive criteria for diagnosing PA using saline infusion test involves an objective interpretation where in the assessors strictly followed the study definition of a positive or negative saline test result.

## Conclusion

In a subset of hypertensive patients with spontaneous hypokalemia who had initial screening test results of PAC > 20 ng/dL and PRA < 0.6 ng/ml/hr may be presumably diagnosed as having overt primary aldosteronism and may not need to proceed with dynamic confirmatory testing. Overall, despite the study limitations, a focused effort to validate the existing simplified confirmatory algorithm may lead to diagnostic efficiency towards improving patient outcomes in relation to prompt diagnosis, cost reduction, addressing limited resources, and further support guideline recommendations. For future studies, larger trials that are prospective in design, inclusion of a wider hypertensive population not restricted to PA screened patients, use of a uniform, standardized assay methodology and blinding of assessors are recommended.

## Data Availability

The datasets used and/or analyzed during the current study are available from the corresponding author on reasonable request.

## Abbreviations

PAC: plasma aldosterone concentration
PRA: plasma renin activity
PA: primary aldosteronism
SIT: saline infusion test
ARR: aldosterone renin ratio
K^+^: potassium
